# Reevaluation of the US Pathologist Workforce Size

**DOI:** 10.1001/jamanetworkopen.2020.10648

**Published:** 2020-07-16

**Authors:** Stanley J. Robboy, David Gross, Jason Y. Park, Elizabeth Kittrie, James M. Crawford, Rebecca L. Johnson, Michael B. Cohen, Donald S. Karcher, Robert D. Hoffman, Anthony T. Smith, W. Stephen Black-Schaffer

**Affiliations:** 1 Department of Pathology, Duke University Medical Center, Durham, North Carolina; 2 College of American Pathologists, Washington, DC; 3 Department of Pathology, University of Texas Southwestern Medical Center, Dallas; 4 Eugene McDermott Center for Human Growth and Development, University of Texas Southwestern Medical Center, Dallas; 5 US Department of Health and Human Services, Health Resources and Services Administration, Bureau of Health Workforce, Rockville, Maryland; 6 Department of Pathology and Laboratory Medicine, Donald and Barbara Zucker School of Medicine at Hofstra/Northwell, Hempstead, New York; 7 American Board of Pathology, Tampa, Florida; 8 Department of Pathology, Wake Forest School of Medicine, Winston-Salem, North Carolina; 9 Department of Pathology, The George Washington University Medical Center, Washington, DC; 10 Department of Pathology, Vanderbilt University Medical Center, Nashville, Tennessee; 11 College of American Pathologists, Northfield, Illinois; 12 Department of Pathology, Massachusetts General Hospital, Harvard Medical School, Boston

## Abstract

**Importance:**

There is currently no national organization that publishes its data that serves as the authoritative source of the pathologist workforce in the US. Accurate physician numbers are needed to plan for future health care service requirements.

**Objective:**

To assess the accuracy of current pathologist workforce estimates in the US by examining why divergency appears in different published resources.

**Design, Setting, and Participants:**

This study examined the American Board of Pathology classification for pathologist primary specialty and subspecialties and analyzed previously published reports from the following data sources: the Association of American Medical Colleges (AAMC), the Accreditation Council for Graduate Medical Education (ACGME), a 2013 College of American Pathologists (CAP) report, a commercially available version of the American Medical Assoication (AMA) Physician Masterfile, and an unpublished data summary from June 10, 2019.

**Main Outcomes and Measures:**

Number of physicians classified as pathologists.

**Results:**

The most recent AAMC data from 2017 (published in 2018) reported 12 839 physicians practicing “anatomic/clinical pathology,” which is a subset of the whole. In comparison, the current AMA Physician Masterfile, which is not available publicly, listed 21 292 active pathologists in June 2019. The AMA Physician Masterfile includes all pathologists in 15 subspecialized training areas as identified by the ACGME. By contrast, AAMC’s data, which derive from the AMA Physician Masterfile data, only count physicians primarily associated with 3 general categories of pathologists and 1 subspecialty category (ie, chemical pathology). Thus, the AAMC pathology workforce estimate does not include those whose principal work is in 11 subspecialty areas, such as blood banking or transfusion medicine, cytopathology, hematopathology, or microbiology. An additional discrepancy relates to the ACGME residency (specialties) and fellowship (subspecialties) training programs in which pathologists with training in dermatopathology appear as dermatologists and pathologists with training in molecular genetic pathology appear as medical geneticists.

**Conclusions and Relevance:**

This analysis found that most sources reported only select categories of the pathologist workforce rather than the complete workforce. The discordant nature of reporting may pertain to other medical specialties that have undergone increased subspecialization during the past 2 decades (eg, surgery and medicine). Reconsideration of the methods for determining the pathologist workforce and for all workforces in medicine appears to be needed.

## Key Points

**Question:**

What is the current size of the US pathologist workforce?

**Findings:**

This analysis found that the American Medical Association’s Physician Masterfile listed 21 292 active pathologists as of June 2019 compared with 12 839 anatomical and clinical pathologists reported by the Association of American Medical Colleges for 2017 and exceeded all previously published estimates in the past decade.

**Meaning:**

Accurate physician workforce assessment for all specialties may require a reexamination of the methods used in producing current and previous estimates.

## Teaser

This analysis assesses the accuracy of current pathologist workforce estimates in the US by examining why divergency appears in different published resources.

## Introduction

Published estimates of the size of the pathology workforce vary considerably. Two analyses^1,2^ revealed the challenges in determining the true supply because there are discrepancies in how the data are captured and the methods used to collect the underlying data. For example, Metter et al^1^ analyzed the trends in the US pathology workforce regarding the number of practicing pathologists in the US (and also Canada) between 2007 and 2017 using data from the Association of American Medical Colleges (AAMC) Physician Workforce Data Books Canadian Medical Association Physician Masterfile for Canada.^3^ Key findings were that the current number of pathologists was only 12 839 and the pathology workforce has been decreasing since at least 2007. These findings conflict with another earlier report^2^ based on data available from commercially sold versions of the American Medical Association (AMA) Physician Masterfile adjusted for physicians who do not permit release of their names (determined by comparison analyses of physicians listed in state medical board registries and the AMA-released Masterfile). That analysis found that the stated number of pathologists in the US in 2010 was substantially greater (approximately 18 000 full-time equivalents) and projected that, because of the reduction of total number of residents in pathology programs since 1975 (maximum, 3600 residents),^4^ the workforce would begin a slow decrease in 2014, reaching a minimum in approximately 2021.

These data sources can be used to examine the discrepancies that exist between workforce determinations for pathology and how national organizations compile and report their data. As part of an ongoing, more extensive analysis of the pathologist workforce, we assessed the accuracy of current pathologist estimates in the US. In addition, we examined how other organizations’ rules influence pathology workforce counts.

## Methods

This analysis examined previously published reports from the following data sources: the AAMC,^5^ the Accreditation Council for Graduate Medical Education (ACGME),^6^ a 2013 College of American Pathologists (CAP) report,^2^ a commercially available version of the AMA Physician Masterfile,^7^ and an unpublished data summary from June 10, 2019, that was provided by the AMA (courtesy of the AMA’s Database Products Portfolio Division).

The data from the AAMC have been given in biennial reports since 2006 and are based on the AMA Physician Masterfile. Since 2000, the AAMC has included additional data from the GME (Graduate Medical Education) Track, which contains the National GME Census data jointly obtained by the AMA and the AAMC. The latest ACGME specialties report is from 2018 and is composed of data collected through 2017. The data from the ACGME are from annual reports from 2002 to the present. The report that the AMA provided to us responded to our specific request (for all active US pathologists) based on the subject of this report. We had no access to query any portion of the underlying files from any of these 3 data sources. This is a descriptive analysis, and no statistical analyses were performed.

## Results

Collection and dissemination of pathology workforce data involve a web of interconnected entities. The AMA serves as the lead, responsible for aggregating data from many different sources into its Physician Masterfile. The ACGME is the principal organization that collects and supplies information on residency (specialty) and subspecialty (fellowship) programs. For practice specialty, the principal data source is the ACGME accredited training (residency and fellowship) programs. The GME Track is a resident database and tracking system introduced in 2000 to assist GME administrators and program directors in the collection and management of GME data.^8^ GME Track contains the National GME Census, which the AAMC and AMA use to reduce duplicative reporting. The American Board of Pathology (ABPath), although not collecting and reporting workforce data, sets the basic rules for what are considered the primary categories for certification as pathologists and the secondary categories for the subspecialties of pathology (eg, dermatopathology).

### AAMC Presentation of AMA Data

The AMA Physician Masterfile includes a primary specialty field that lists the principal specialty or subspecialty of all physicians in the US. The data populating it are principally derived from the ACGME (residency and fellowship training) but also incorporate information that individual physicians may report to the AMA. Each physician may only be associated with 1 primary specialty. For consistency, *specialty* refers to the field of pathology and *subspecialty* to any of its parts (eg, dermatopathology). The primary specialty field has 15 possible unique entries for pathologists (Box), but only 1 is assigned per pathologist (a subspecialty counts for purposes of this field). The most recently recorded specialty or subspecialty for a pathologist automatically replaces prior entries. The primary specialty field is used only for workforce counting.

Box. **Specialty Classifications**

**ABPath**

**Primary**

Anatomic/clinical pathology
Anatomic pathology
Clinical pathology
Anatomic pathology/neuropathology

**Subspecialties**

Blood banking/transfusion medicine
Chemical pathology
Clinical informatics
Cytopathology
Dermatopathology
Forensic pathology
Hematology
Medical microbiology
Molecular pathology
Neuropathology
Pediatric pathology

**AAMC**

Anatomic pathology
Anatomic/clinical pathology
Chemical pathology
Clinical pathology
For reporting, the AAMC recognizes only the above

**ACGME**

Anatomic/clinical pathology

**Subspecialties**

Blood banking/transfusion medicine
Chemical pathology
Clinical informatics (pathology) (since 2015)
Cytopathology
Forensic pathology
Hematology (pathology)
Medical microbiology
Neuropathology
Pediatric pathology
Selective pathology

**Ambiguous**

Anatomic pathology (legacy designation)
Clinical pathology (legacy designation)
Anatomic pathology/neuropathology (classified as subspecialty neuropathology)
Dermatopathology (multidisciplinary but listed under dermatology)
Molecular genetic pathology (multidisciplinary since at least 2007 but listed under medical genetics and genomics)

**AMA (Based on ACGME)**

Anatomic/clinical pathology
Anatomic pathology
Blood banking/transfusion medicine
Chemical pathology
Clinical informatics (pathology) (since 2015)
Clinical pathology
Cytopathology
Dermatopathology (pathology)
Forensic pathology
Hematology (pathology)
Medical microbiology
Molecular genetic pathology (pathology and medical genetics)
Neuropathology
Pediatric pathology
Selective pathology

Abbreviations: AAMC, Association of American Medical Colleges; ABPath, American Board of Pathology; ACGME, Accreditation Council for Graduate Medical Education; AMA, American Medical Association.

Beginning in 2006, the AAMC enumerated pathologists with the descriptor “anatomic/clinical pathology” to encompass the ABPath categories of anatomic pathology, clinical pathology, anatomic/clinical pathology, and a single subspecialty (chemical pathology).^9,10^ The AAMC workforce data do not include other pathology subspecialties recognized by the AMA and ACGME (Box). Thus, when a pathologist has a subspecialty other than chemical pathology as their AMA Physician Masterfile primary specialty, the AAMC does not count that individual. Using the commercially available Masterfile database, which lacks members who do not wish their names released and which we have corrected by using state medical registries, we estimated the pathology workforce in 2013 to be 17 968. Review of earlier AAMC reports showed that these designations have been in place for many years. The rationale for the AAMC method is not provided. Because the AMA does not report workforce numbers publicly, the only publicly available data from the AMA Physician Masterfile is that accessible through the AAMC biennial reports.

The AMA Database Products Division provided us with the current numerical summaries of the pathologist workforce, including all subspecialty categories (Table 1). As of June 10, 2019, the AMA listed 21 292 active pathologists. On the basis of the same method, the number of active pathologists, exclusive of semiretired and retired pathologists and residents, was 18 822 in 2011, 19 606 in 2014, and 19 923 in 2017 (courtesy of the AMA Database Products Division from past years). The AAMC, using the received AMA data (2017 physician data were provided in the most recent 2018 biennial report), listed only 12 839 active anatomic/clinical pathologists, a subset of the whole (Table 2). The remaining pathologists were not counted because they fall into pathology subspecialties not included in the AAMC methods.

**Table 1.** American Medical Association Listing of All Pathologists (Without Residents and Retirees)

Specialty description	Pathologists, No.									
	Administration	Full-time hospital staff	Locum tenens	Medical teaching	Not classified	Office-based practice	Other	Research	Semiretired	Total
Anatomic pathology	29	257	2	46	15	359	64	116	30	918
Blood bank	75	130	0	21	183	298	26	22	10	765
Clinical informatics (pathology)^a^	1	1	0	0	4	5	2	0	0	13
Clinical pathology	88	72	0	14	4	102	26	70	23	399
Dermatopathology	3	124	0	24	159	717	63	9	1	1100
Forensic pathology	23	96	0	12	155	441	159	3	31	920
Hematology (pathology)	4	221	1	38	439	711	44	10	3	1471
Molecular (genetic) pathology	3	22	1	21	169	87	7	9	2	321
Medical microbiology	6	18	0	7	34	37	3	6	1	112
Neuropathology	5	60	1	37	121	148	10	49	8	439
Chemical pathology	4	1	0	2	7	9	4	2	1	30
Cytopathology	3	263	0	59	488	769	30	3	3	1618
Pediatric pathology	0	61	0	13	96	86	7	1	4	268
Anatomic/clinical pathology	331	2394	19	328	1761	5705	641	342	284	11 805
Selective pathology^b^	NA	128	NA	44	485	431	20	4	1	1113
Total	575	3848	24	666	4120	9905	1106	646	402	21 292

Abbreviation: NA, not applicable.

^a^ The American Board of Pathology and a second American Board may certify the same subspecialty. The numbers provided are only for those candidates the American Board of Pathology has certified.

^b^ Selective pathology is not an American Board of Pathology–certified subspecialty but indicates when a pathologist has successfully completed a recognized fellowship training program in a subspecialty (eg, general surgical pathology or gastrointestinal surgical pathology) that the Accreditation Council for Graduate Medical Education reviews.

**Table 2.** Sources of Pathologist Workforce Numbers

Source	Pathologists, No.	Date of data collection	Data source	Comment
AMA, 2019	21 292	June 2019	AMA	Data private
AMA Physician Masterfile, commercially available 2018	19 222	October 2018	AMA licensee	Masterfile available through database licensee
AAMC report^11^ and Metter et al,^1^ 2019	12 839	2017	AAMC and AMA	Released biennially. omits all pathologists with specialty boards
Fraher et al^12^	Approximately 17 700	June 2013	Unclear	Likely from AMA commercial datafile. assumes an approximately 2% growth per year
Robboy et al,^2^ 2013 and Robboy et al,^13^ 2015	17 986	December 2010	Multiple	Based on AMA commercially available Masterfile emended after state medical boards comparative review

Abbreviations: AAMC, Association of American Medical Colleges; AMA, American Medical Association.

### Workforce Correlations

The data summary provided by the AMA (Table 1) indicates a count of 7731 pathologists listed by subspecialty if including selective pathology as a subspecialty (total of all lines minus anatomic pathology, clinical pathology, and anatomic/clinical pathology). Once this discrepancy was accounted for, the difference between the AAMC/AMA database and the AMA database alone decreased (8453 vs 7731). The small variance was plausible considering that the 2 databases were nearly 2 years apart. In addition, some of the difference may be attributable to how the AAMC, AMA, and insurance companies treat the status of fellows; fellows in non–ACGME-approved programs can sometimes bill in their own names and may be considered fully active physicians.

### ACGME Misclassification of Some Subspecialties

We found that the annual ACGME Data Resource Books^14^ anomalously report 2 pathology subspecialist categories. Dermatopathology and molecular pathology are both subspecialties in which 2 different boards issue certificates. The ACGME Data Resource Books^14^ list all dermatopathologist trainees under the dermatology section in part because it is unknown during training whether the ABPath or American Board of Dermatology will issue the categorical specialty certificate. Similarly, all molecular pathologists appear under medical genetics, whether granted by the ABPath or the American Board of Medical Genetics and Genomics. According to the ABPath, since 2004, a total of 61% of the 1166 dermatopathology trainees have been pathologists and that 99.8% of the 391 molecular pathology trainees have been pathologists (R.J., written communication, May 2020).

## Discussion

The primary data source for nearly all US physician workforce analyses has been the AMA Physician Masterfile. The AMA, in maintaining the most definitive physician workforce database in the US, uses multiple sources to update the Masterfile. For practice specialty, the principal data source is from ACGME-accredited training (residency and fellowship) programs and now, together with the AAMC, the GME Track. Individuals may at any time also update their AMA record and self-declare themselves to be pathologists regardless of ACGME-accredited training or ABPath board certification.

The AMA, which in past years published the *Physician Characteristics and Distribution in the US*,^15^ does not currently publish these volumes. The AMA Physician Masterfile data can be licensed from third-party vendors, but such sets lack physicians who do not want their names released. The vendors also are authorized to make additional changes and customize the databases responsive to specific requests from third parties. For example, names of semiretired and retired physicians or active physicians who want commercial entities not to contact them may be eliminated.

Although the ABPath plays no role in the workforce enumeration, it is the sole organization charged for setting the examination standards for certification as a pathologist and, in this role, has established the categories for primary certification and for certification in all major subspecialties. A requirement for all applicants is to have completed a GME program in pathology or a pathology subspecialty that the ACGME accredits. All pathologists must obtain a primary certificate in 1 of 4 areas and, with additional training, may receive additional certificates in 1 or more of the subspecialties listed in the Box.

The ACGME, the AMA (which uses the ACGME classification terms), and the AAMC follow the ABPath classification closely but not exactly. The ACGME lists pathology in its annual report as a specialty with 10 subspecialties. For the primary specialty, it reports only a single area: anatomic/clinical pathology. It is uncertain how anatomic pathology and clinical pathology are reported. The ACGME includes selective pathology as a subspecialty, even though there is no ABPath board certification for these areas.

The AMA uses only 3 of the 4 ABPath areas of primary certification (Box). The AMA also added 1 new specialty area for which there is no ABPath board certification: selective pathology.

The AAMC does not count the specialty of pathology as a whole but rather enumerates only a single subset (anatomic/clinical pathology) and, for reporting purposes, includes in this term 4 areas (Box). In contrast to the ABPath, the AAMC categorizes chemical pathology as a primary area of endeavor and does not categorize the ABPath’s category of anatomic pathology/neuropathology as a primary specialty. The AAMC does not report data at the subspecialty level and does not recognize that its data source, the AMA Physician Masterfile, replaces the pathology primary specialty with the latest reported training. Thus, if an anatomic/clinical pathologist receives later training in hematopathology, the physician’s primary specialty field is replaced with the subspecialty hematopathology and the physician is no longer counted by the AAMC as a pathologist.

The most recent AAMC data^16^ (through 2017 and published in 2018) reported that the total active pathologist workforce, which it presents as anatomic/clinical pathology, included 12 839 individuals (9130 in patient care, 388 in teaching, 528 in research, and 2793 categorized as other) (Table 2). This number contrasts with that reported in a prior analysis^2^ of workforce supply and in the summary data on membership that the AMA provided privately to the CAP in the past. The discrepancy was attributable to an erroneous interpretation made by workforce researchers that the AAMC reports only a subset of the profession.^17^ The earlier analysis^2^ used data that did not come directly from the AMA but rather was from a commercial vendor whose Masterfile was limited by exclusion of physicians who did not want their names released. We partially corrected the number by identifying the discrepancies between the AMA counts and by a detailed count of pathologists listed in searchable individual state medical workforce databases.

The 40% difference between the AMA count (n = 21 292) and the AAMC’s most recent report (n = 12 839) is substantial; however, the AAMC count includes only a subset of the pathology workforce. We recommend that the AAMC or any other organization relying on these data alters how it reports the pathology workforce and includes all individuals in the AMA Physician Masterfile list as pathologists. We recommend that further previously published reports on pathology workforce based on AAMC information be reexamined.^1,9,10,11,18,19,20^

Similarly, the criteria that the AAMC and ACGME use to consider a physician as a pathologist and as a subspecialist should be reexamined to help clarify their files and reports. The ACGME updates information about US graduates in training programs for residency and fellowships. In addition, differences exist among those who partook only in a portion of the residency or fellowship program, those who graduated from the program, and those who attained board certification.

Metter et al,^1^ using the AAMC publications, concluded that the US pathologist workforce steadily decreased from 2007 to 2019. All of us made the same error before recognizing that the AAMC reported only pathologists with a database category of anatomic/clinical pathology and not the specialty as a whole. To the surprise of many researchers, the most recent AAMC report^11^ reported that anatomic/clinical pathology had the second greatest workforce decrease (8.3%) of any physician specialty between 2012 and 2017 and cited the AMA Physician Masterfile as its source. This was an artifact of the data used for comparison. In reality, the current AMA Physician Masterfile suggests that the pathologist workforce may be increasing, although strictly comparable reanalysis of prior years’ data is required to form a conclusion. Additional research is needed to clarify the current AMA Physician Masterfile method, including the listing of physicians by self-declaration rather than documented training.

On the basis of the annual ACGME Data Resource Books for academic years 2002 through 2018, an increasing proportion of pathologist trainees took specialty fellowships during those years and thus have been excluded from being counted as pathologists by the AAMC method. It is likely that the increasing percentage (now 97%) of new-in-practice pathologists with subspecialty training has resulted in the decreasing number of practitioners in the AAMC reports. A future area of work to document the actual changes in the workforce over multiple periods is needed, specifically comparing numbers of trainees with subspecialty training entering the profession with those retiring.

We believe that the situation we are describing for pathology may also be occurring in general surgery. Surgeons with general surgery training can subspecialize in multiple areas (this is why many centers have subspecialized pathologists). General surgeons can undergo focused fellowship training and subspecialize in areas such as breast, gastrointestinal, sarcoma, hepatobiliary, and transplant surgery. If a surgeon undergoes a subspecialty fellowship or lists his or her primary category in the AMA Physician Masterfile as a breast surgeon, sarcoma surgeon, or hepatobiliary surgeon, how does the AAMC method count them? Thus, the 2012 *AAMC Databook* lists 26 314 active general surgeons, but in the 2017 *AAMC Databook*, 25 042 general surgeons are listed.^21^ This finding raises the question: Does the change represent a real decrease, or is it an artifact of the counting and classification methods?

The data from the ACGME reports may also affect the workforce count. Although the ACGME is unable to distinguish which board will eventually certify each trainee, the organization has historically assigned all dermatopathologists to dermatology and all molecular pathologists to medical genetics even though, based on ABPath data, since 2004 a total of 61% of the 1166 dermatopathology trainees and 99.8% of the 391 molecular pathology trainees have been pathologists. We believe it would be more useful and appropriate to assign these trainees to the specialty in which most are certified.

Accurate workforce numbers have many different applications. At a most basic level, the fee that the ABPath must pay annually for membership to the American Board of Medical Specialties depends on the number of pathologists in practice. From the perspective of students considering medicine as a profession or the type of residency to choose later, questions of specialty viability and job availability are paramount. Inaccurate or incomplete depictions of the existing or projected supply of physician subspecialties can misleadingly affect career choices.

Tools for modeling the workforce are vital when planning for the future demand.^13^ The AAMC is developing a modeling tool to improve analytic capabilities with regard to changing physician work patterns, market saturation and displacement of occupations and select specialties, current shortages and inefficiencies, and new care delivery and financing models.^22^ The modeling tool that the CAP developed^23^ has been useful in evaluating future workforce needs for subspecialties (eg, molecular diagnostics).^13^ Fraher et al^12,24^ currently have the only online tool available for public use. It permits workforce examination at the state level and even at the level of small geographic zones. Such innovative tools are useful for planning workforce because they are interactive, web based, and user friendly. They give users the ability to play “what if” scenarios and help estimate where shortages or surpluses will occur. However, reliable and complete data are imperative if these modeling tools are to provide meaningful insights.

### Limitations

This study has limitations. We did not have access to the data behind the reports; thus, individual physicians listed in each organization’s database could not be identified and matched and inconsistencies may have occurred. Considerations of privacy and Health Insurance Portability and Accountability Act may be obstacles to these data becoming available.

## Conclusions

Workforce counts are important but often complicated to execute. Without them, intelligent planning for the future becomes impossible. This current analysis suggests that reevaluation of the current AAMC and ACGME methods are needed not only for pathology but likely for all medical specialties. We believe that accurate workforce data are crucial for proper health care service planning.

## References

[1] Metter DM, Colgan TJ, Leung ST, Timmons CF, Park JY Trends in the US and Canadian pathologist workforces from 2007 to 2017. JAMA Netw Open. 2019;2(5):e194337. doi:10.1001/jamanetworkopen.2019.4337 31150073 PMC6547243

[2] Robboy SJ, Weintraub S, Horvath AE, Pathologist workforce in the United States, I: development of a predictive model to examine factors influencing supply. Arch Pathol Lab Med. 2013;137(12):1723-1732. doi:10.5858/arpa.2013-0200-OA 23738764

[3] Association of American Medical Colleges 2017 State Physician Workforce Data Book. Accessed March 28, 2019. https://www.aamc.org/data/workforce/reports/484392/2017-state-physician-workforce-data-report.html

[4] Alexander CB Pathology graduate medical education (overview from 2006-2010). Hum Pathol. 2011;42(6):763-769. doi:10.1016/j.humpath.2010.11.008 21333325

[5] Association of American Medical Colleges Accessed June 13, 2020. https://www.aamc.org/

[6] Accreditation Council for Graduate Medical Education Accessed June 13, 2020. http://www.acgme.com/

[7] American Medical Association AMA Physician Masterfile. Accessed June 7, 2020. https://www.ama-assn.org/practice-management/masterfile/ama-physician-masterfile

[8] GME Track. Secondary GME Track 2019 Accessed January 27, 2020. https://www.aamc.org/data-reports/students-residents/report/gme-track

[9] Erikson C 2014 Physician Specialty Data Book. Center for Workforce Studies; 2014.

[10] Erikson C, Jones K, Tilton C 2012 Physician Specialty Data Book. Center for Workforce Studies; 2012.

[11] Association of American Medical Colleges. 2018 Physician Specialty Data Book, Center for Workforce Studies. 2018 Accessed June 13, 2020. https://www.aamc.org/data-reports/workforce/interactive-data/active-physicians-sex-and-specialty-2017

[12] Fraher E, Knapton A, Holmes GM FutureDocs Forecasting Tool. Cecil G. Sheps Center for Health Services Research, University of North Carolina-Chapel Hill; 2016.

[13] Robboy SJ, Gupta S, Crawford JM, The pathologist workforce in the United States, II: an interactive modeling tool for analyzing future qualitative and quantitative staffing demands for services. Arch Pathol Lab Med. 2015;139(11):1413-1430. doi:10.5858/arpa.2014-0559-OA 26516939

[14] Accreditation Council for Graduate Medical Education, Data Resource Book Accessed January 3, 2020. https://www.acgme.org/About-Us/Publications-and-Resources/Graduate-Medical-Education-Data-Resource-Book 2018-2019

[15] American Medical Association Physician Characteristics and Distribution in the US. American Medical Association; 2008.

[16] Association of American Medical Colleges Table 1.1. Number of active physicians in the largest specialties by major professional activity, 2017 In: *Active Physicians in the Largest Specialties* Association of American Medical Colleges; 2017. Accessed June 7, 2020. https://www.aamc.org/data-reports/workforce/interactive-data/active-physicians-largest-specialties-2017

[17] Bowden A New data book reports increase in Iowa’s physician workforce. 2017 Accessed June 7, 2020. https://medicalboard.iowa.gov/sites/default/files/documents/2018/04/press_release_-_new_data_book_shows_increase_in_iowa_physician_workforce_-_december_29_2017.pdf

[18] Yamagata H *AAMC Physician Specialty Data** (*2006*): **A Chart Book.* Center for Workforce Studies; 2006.

[19] Salsberg ES, Rivers KL 2008 Physician Specialty Data. Center for Workforce Studies; 2008.

[20] Association of American Medical Colleges. 2016 Physician Specialty Data Book, Center for Workforce Studies. 2016 Accessed June 13, 2020. https://www.aamc.org/data-reports/workforce/interactive-data/active-physicians-sex-and-specialty-2015

[21] Ellison EC, Pawlik TM, Way DP, Satiani B, Williams TE Ten-year reassessment of the shortage of general surgeons: increases in graduation numbers of general surgery residents are insufficient to meet the future demand for general surgeons. Surgery. 2018;164(4):726-732. doi:10.1016/j.surg.2018.04.042 30098811

[22] Dall T, Reynols R, Jones K, Chakrabarti R, Iacobucci W The complexities of physician supply and demand: projections from 2017 to 2032. 2019 Accessed July 7, 2019. https://aamc-black.global.ssl.fastly.net/production/media/filer_public/31/13/3113ee5c-a038-4c16-89af-294a69826650/2019_update_-_the_complexities_of_physician_supply_and_demand_-_projections_from_2017-2032.pdf

[23] Gupta S, Black-Schaffer WS, Crawford JM, An innovative interactive modeling tool to analyze scenario-based physician workforce supply and demand. Acad Pathol. 2015;2(4):2374289515606730. doi:10.1177/2374289515606730 28725751 PMC5479464

[24] Fraher E. Health workforce modeling: past, present and future challenges and opportunities. Paper presented at: Association of American Medical Colleges Physician Workforce Research Conference; May 3, 2013; Washington, DC.

